# Are the Cognitive Alterations Present in Children Born From Preeclamptic Pregnancies the Result of Impaired Angiogenesis? Focus on the Potential Role of the VEGF Family

**DOI:** 10.3389/fphys.2018.01591

**Published:** 2018-11-14

**Authors:** Evelyn Lara, Jesenia Acurio, José Leon, Jeffrey Penny, Pablo Torres-Vergara, Carlos Escudero

**Affiliations:** ^1^Vascular Physiology Laboratory, Group of Investigation in Tumor Angiogenesis (LFV-GIANT), Department of Basic Sciences, Faculty of Basic Sciences, Universidad del Bío-Bío, Chillán, Chile; ^2^Group of Research and Innovation in Vascular Health (GRIVAS Health), Chillán, Chile; ^3^Division of Pharmacy and Optometry, School of Health Sciences, Faculty of Biology, Medicine and Health, The University of Manchester, Manchester, United Kingdom; ^4^Department of Pharmacy, Faculty of Pharmacy, Universidad de Concepción, Concepción, Chile; ^5^Red Iberoamericana de alteraciones Vasculares Asociadas a TRastornos del EMbarazo (RIVA-TREM), Chillán, Chile

**Keywords:** preeclampsia, angiogenesis, neurovascular, neurocognitive, vascular endothelial growth factor, placental growth factor, sFlt-1

## Abstract

Evidence from clinical studies has proposed that children born from preeclamptic women have a higher risk of suffering neurological, psychological, or behavioral alterations. However, to date, the mechanisms behind these outcomes are poorly understood. Here, we speculate that the neurodevelopmental alterations in the children of preeclamptic pregnancies result from impaired angiogenesis. The pro-angiogenic factors vascular endothelial growth factor (VEGF) and placental growth factor (PlGF) are key regulators of both vascular and neurological development, and it has been widely demonstrated that umbilical blood of preeclamptic pregnancies contains high levels of soluble VEGF receptor type 1 (sFlt-1), a decoy receptor of VEGF. As a consequence, this anti-angiogenic state could lead to long-lasting neurological outcomes. In this non-systematic review, we propose that alterations in the circulating concentrations of VEGF, PlGF, and sFlt-1 in preeclamptic pregnancies will affect both fetal cerebrovascular function and neurodevelopment, which in turn may cause cognitive alterations in post-natal life.

## Introduction

Preeclampsia is a multisystemic syndrome of unknown etiology that affects pregnant women after 20 weeks of gestation. The clinical presentation of preeclampsia is characterized by hypertension with co-morbidities including proteinuria or alterations in coagulation and liver function, thrombocytopenia, pulmonary edema, and brain or visual impairments (ACOG, 2013). The global incidence of preeclampsia ranges from 2 to 8% of all pregnancies, with the condition being the leading cause of maternal morbidity and mortality, premature birth and perinatal death in both developed and developing countries (Duley, 2009). Furthermore, 30% of newborns to preeclamptic mothers experience some form of adverse perinatal outcome, including prematurity and intrauterine growth retardation among others (Villar et al., 2006). Although the remaining percentage (∼70%) of newborns may be considered healthy, evidence suggests that children born to preeclampsia have a higher risk of suffering cardiovascular-related diseases compared to children born to normal pregnancies (Davis et al., 2012a).

The etiology of preeclampsia is not well known, but the most accepted hypothesis targets the placenta as a primary source of endogenous factors that, upon release to the systemic circulation, impair the functionality of endothelial cells. Previous reports have proposed that anti-angiogenic factors, including the soluble fms-1-like tyrosine kinase-1, (sFlt-1 or sVEGFR-1) and soluble endoglin (sENG) are responsible for the endothelial dysfunction present in preeclampsia (see details in Roberts and Escudero, 2012).

Furthermore, multiple studies have shown alterations in the balance of pro-angiogenic and anti-angiogenic factors in the placenta at term, in the fetal-placental circulation, and in the circulation of preeclamptic women and their children years after the preeclamptic incident (Maynard and Karumanchi, 2011; Rana et al., 2014; Karumanchi, 2016).

In addition to imbalances in pro and anti-angiogenic processes, endothelial dysfunction is a well-recognized feature in the offspring born to preeclampsia, the consequences of which include cardiovascular disease in adulthood (Davis et al., 2012a,b). In this manuscript, we extend this concept to propose that impaired angiogenesis affects the cognitive development of children born from preeclamptic women.

## Evidence of Cognitive Alterations in the Offspring Born to Preeclamptic Pregnancies

The pathophysiology of pregnancy-related disorders such as preeclampsia is associated with impaired placental function that compromises the interaction between mother and fetus (Barker, 1995; Krause et al., 2009; Escudero et al., 2014b; Thornburg, 2015). These alterations in the mother-fetus interaction apparently generate adaptive changes in the functionality of the endocrine, metabolic, vascular and immune systems of the fetus, with consequences in post-natal life. In this regard, several reports have proposed that the offspring of preeclamptic pregnancies have a higher risk of developing metabolic, neurological and cardiovascular disorders in adulthood (Tenhola et al., 2003; Wu et al., 2008, 2009, 2011; Robinson et al., 2009; Jayet et al., 2010; Davis et al., 2012a; Tuovinen et al., 2012; Escudero et al., 2014b; Alsnes et al., 2016; Pinheiro et al., 2016; Ratsep et al., 2016a,b; Figueiro-Filho et al., 2017a).

At a cognitive level, several studies have described that children born to preeclamptic women exhibit an increased risk of developing cerebral palsy (Szymonowicz and Yu, 1987), cerebral stroke (Kajantie et al., 2009), impaired neurological development (Spinillo et al., 1994), developmental delays at the age of 5 years (Warshafsky et al., 2016), poor cognitive development (Cheng et al., 2004), intellectual disability (Griffith et al., 2011), anxiety (Tuovinen et al., 2012), depressive symptoms (Tuovinen et al., 2010), attention deficit disorder and hyperactivity (Getahun et al., 2013), and other mental disorders (Tuovinen et al., 2014) in comparison with children born to normotensive women. In agreement with this body of evidence, a recent systematic review concluded that preeclampsia is associated with neurocognitive alterations in children (Figueiro-Filho et al., 2017b).

## Brain Alterations in Offspring Born to Preeclampsia

Pre-clinical studies have attempted to establish an explanation for the variety of pathoneurological consequences associated with preeclampsia. Studies report that the brain weight at birth of the offspring born to hypertensive pregnant rats was significantly lower compared to the offspring born to their normotensive counterparts (Liu et al., 2016). Furthermore, reduced brain weight was associated with reduced expression of markers of neurogenesis, markers of neuroproliferation in the cortex and with severe impairments in spatial learning and memory.

In the clinical setting, Dr. Ana Croy’s group have been conducting pioneering studies which attempt to correlate cognitive parameters with brain anatomy (Ratsep et al., 2016a,c) or neuronal networking (Mak et al., 2018). The findings of these magnetic resonance imaging (MRI) studies, in 10 children born to preeclampsia and 10 children born to normotensive pregnancies, demonstrated that at least five brain regions, including the cerebellum, temporal lobe, brainstem, and right and left amygdala, were larger in children born to preeclampsia compared with controls (Ratsep et al., 2016c). The study also demonstrated cerebral blood flow in both parietal and occipital lobes in children born to preeclamptic women was lower than in controls. The authors proposed that changes in brain vasculature may have preceded the structural alterations. Consistent with these findings, the MRI studies of Figueiro-Filho et al. (2017a) reported increased volumes of the tract for the superior longitudinal fasciculus and the caudate nucleus, amongst other alterations, in the brains of children born to preeclamptic pregnancies.

More recently, a study suggested that children born from preeclamptic pregnancies demonstrated higher levels of connectivity between the left amygdala and bilateral frontal pole; the right amygdala and the left frontal pole, and between the medial prefrontal cortex and precuneus, compared to matched-control children (Mak et al., 2018). Furthermore, this study also found that children born from preeclamptic pregnancies exhibit decreased connectivity between the medial prefrontal cortex and the left occipital fusiform gyrus.

The body of findings available therefore provides strong, albeit preliminary, evidence for the hypothesis that the neurological and cognitive impairments present in children delivered from preeclamptic pregnancies are likely the result of neurodevelopmental alterations. Furthermore, complementary to this hypothesis is the fact that impaired vascular development in the fetal brain could be a contributing factor to preeclampsia-associated impairments.

Crucially, a more in-depth understanding of (i) the potential confounding factors which impact fetal development, such as altered placental functionality (hypoxia), (ii) subject-specific data, including the weight of the newborn, gestational age at birth and the socioeconomic and nutritional context in which the newborn develops, and (iii) the physiological and morphological modifications in the cerebral vasculature of children from preeclamptic women, will be key to strengthening this hypothesis.

## Evidence of Alterations in the Vasculature of Children Born to Preeclamptic Women

Angiogenesis, defined as the formation of new vessels from an existing vessel, is widespread during fetal and neonatal development, although it is rarely observed in adults under normal conditions (Carmeliet and Ruiz de Almodovar, 2013; Watson et al., 2016, 2017). Although several processes drive vascular development, angiogenesis is one of the most studied in the context of preeclampsia. Conversely, vascular remodeling or “vascular pruning,” the process by which pro-angiogenic signaling is suppressed, resulting in accelerated apoptosis of endothelial cells (Watson et al., 2017) and organized loss of blood vessels, is also observed in the fetus. Consequently, angiogenesis and vascular remodeling are complementary processes, the relative extents of which can influence vascular development not only in the fetuses of normotensive and preeclamptic pregnancies, but can impact vascular architecture in neonates, and in individuals in later life.

Although studies have investigated the characteristics of accessible vascular networks in individuals born to preeclamptic pregnancies, no studies report on how preeclampsia influences the anatomy and physiology of the blood brain barrier in neonates, children, and adults.

Our research group (Escudero et al., 2013, 2014a; Acurio et al., 2014, 2016) and others (Staff et al., 2005; Tsao et al., 2005; Stark et al., 2009; Antonios et al., 2012; Touwslager et al., 2012; Yu et al., 2016) have studied how angiogenesis may be impaired in the children born to preeclamptic pregnancies. Interestingly, it has been shown that full-term babies of hypertensive pregnancies (including preeclampsia) exhibited a reduction in the maximum capillary density per square millimeter on the plantar surface of the big toe compared with control infants (Antonios et al., 2012).

The above results are in agreement with previous findings (Stark et al., 2009; Touwslager et al., 2012) that demonstrated structural and functional alterations in the microvasculature of newborns and increased vascular remodeling in the skin of 3-month old babies born to hypertensive pregnancies (Yu et al., 2016). Also, it is reported (Yu et al., 2012) that children born to preeclampsia exhibited a 45% reduction in the risk of prematurity-associated retinopathy, a condition characterized by increased retinal angiogenesis. Furthermore, studies report the retinal arteriolar caliber, but not the retinal venular caliber, was narrower in school-age children from hypertensive pregnancies, including preeclamptic pregnancies, than in children born from normotensive pregnancies (Yesil et al., 2016). Since prematurity-associated retinopathy is a condition characterized by an increase in the formation of blood vessels in the retina, differences in retinal arteriolar caliber are suggestive of differences in vascular remodeling in the two cohorts.

**FIGURE 1:**
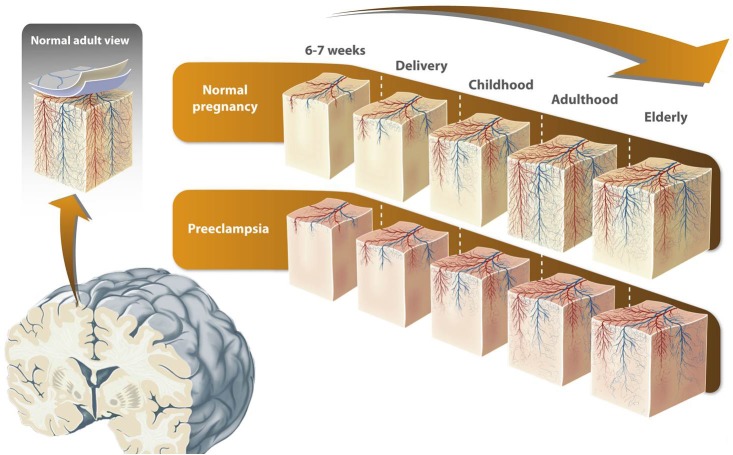
Proposed model of alterations in brain vascular development in offspring born to preeclampsia. Vascular system components in the cerebral cortex emerge directly or indirectly from the pial capillary anastomotic plexus located in the pial lamella (the inner meningeal compartment). The microvascular compartment in the brain cortex is a key component not only for the blood brain barrier but also for cortex development itself. The process of microvasculature formation in the human brain cortex, in which both new vessel formation (angiogenesis) and vascular remodeling takes place, is highly dynamic and not totally understood. Vessel formation in the brain cortex starts during fetal development and continues in the post-natal stage. It is proposed that in the elderly, cortical vessel formation decreases and/or vascular remodeling increases. Since preeclampsia is characterized by an imbalance in pro and anti-angiogenic markers, it has been speculated that cerebral angiogenesis is altered in children born to mothers with preeclampsia. This alteration might be present throughout life, and may explain the greater risk of cognitive alterations in these individuals. The figure is reproduced with permission from the copyright holder.

However, as far as we are aware, although Ratsep et al. (2016c) demonstrated alterations in blood flow to the parietal and occipital lobes in the offspring of preeclamptic pregnancies, the effects of preeclampsia on blood brain barrier anatomy and physiology in neonates, children, and adults are yet to be established. In this manuscript we propose that cerebral angiogenesis is altered in children born to mothers with preeclampsia, thereby initially influencing the early neonatal stage and subsequently throughout life (Figure 1).

Since preeclampsia-associated alterations in vascular networks (Stark et al., 2009; Antonios et al., 2012; Touwslager et al., 2012; Yesil et al., 2016; Yu et al., 2016), neurocognition (Wu et al., 2009; Dang et al., 2016; Ratsep et al., 2016a) and neuroanatomy (Ratsep et al., 2016a,b,c; Figueiro-Filho et al., 2017a; Mak et al., 2018) have been reported, there is an obvious need for an improved understanding of the molecular mechanisms underpinning such physiological changes. Vascular endothelial growth factor (VEGF), placental growth factor (PlGF) and soluble fms-like tyrosine kinase (sFlt-1) play key roles in angiogenesis and neurogenesis (Ruiz de Almodovar et al., 2009; Carmeliet and Ruiz de Almodovar, 2013) and have therefore been extensively studied in preeclampsia. These important biomarkers are discussed in more detail in the next section.

## VEGF and Angiogenesis and Neurogenesis

The VEGF family of proteins is one of the key regulators of both angiogenesis and neurogenesis (Carmeliet and Ruiz de Almodovar, 2013). This family is comprised of five members including VEGF-A, VEGF-B, VEGF-C, VEGF-D, and PlGF, which activate vascular endothelial growth factor receptors (VEGFRs), namely VEGFR-1, VEGFR-2, and VEGFR-3, a group of membrane receptors possessing tyrosine kinase activity. Once activated these receptors mediate several processes including cell migration, proliferation and survival (Shibuya, 2013). In addition, VEGFRs are involved in development of the morphological features of blood vessels and regulate vascular permeability (Olsson et al., 2006). The activity of VEGF and PlGF is regulated by sFlt-1, a circulating variant of VEGRF-1 that prevents their binding to their membrane receptors, thus decreasing their activity (Ahmad et al., 2011). Deletion of VEGF (Carmeliet et al., 1996), VEGFR1 (Fong et al., 1995) or VEGFR2 (Shalaby et al., 1995), or even tyrosine residues (Y1175) responsible for VEGFR2 activation (Sakurai et al., 2005) causes severe failure in angiogenesis, and are embryonic lethal in mice. Furthermore, mice deficient in VEGFR1 die at the embryonic stage due to hypertrophy and disorganization of blood vessels (Fong et al., 1995), rather than a lack of vessel formation.

Several reports have demonstrated that in preeclampsia there is an increase in circulating levels of placental derived sFlt-1 (and other anti-angiogenic molecules such as sENG), thereby generating a state of systemic vascular dysfunction in the mother (see details in Karumanchi, 2018).

**FIGURE 2:**
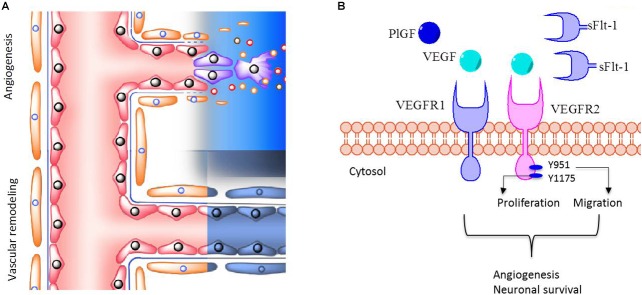
VEGFR1 and VEGFR2 in brain vessel formation. **(A)** The extent of perfusion of brain tissue depends on both angiogenesis and vascular remodeling processes, which are mediated by pro- and anti-angiogenic factors. **(B)** VEGF and PlGF activate VEGFR1 and VEGFR2. Phosphorylation of tyrosine 951 (Y951) or Y1175 is associated with activation of VEGFR2, leading preferentially to cell migration or cell proliferation, respectively. These regulatory mechanisms are involved in angiogenesis and neuronal survival. In contrast, sFlt-1 prevents the binding of VEGF and PlGF to VEGFRs, thus decreasing their pro-angiogenic and neuronal survival activity. In this manuscript it is postulated that children born to preeclampsia exhibit a reduced number of blood vessels in the brain cortex, through impaired angiogenesis and/or increased vascular remodeling. We also propose that the underling mechanism might involve dysregulation in VEGFR2 activation due to high sFlt-1 circulating levels in preeclampsia.

VEGF-mediated activation of VEGFR2 leads to cell migration, proliferation and angiogenesis (Koch and Claesson-Welsh, 2012; Figure 2A), and activation is mediated by phosphorylation of the tyrosine 951 (Y951) and tyrosine 1175 (Y1175) residues. Consequently, alterations in angiogenesis may well be a multi-factorial process, influenced not only by circulating levels of ligand and levels of receptor expression, but by actual kinase-mediated phosphorylation of the receptor itself. Few publications have described activation of VEGFR2 (i.e., tyrosine phosphorylation) in preeclampsia, the findings of which are conflicting (Ahmad and Ahmed, 2004; Escudero et al., 2014a).

Activation of VEGFRs has also been linked with neural development (Ruiz de Almodovar et al., 2009; Carmeliet and Ruiz de Almodovar, 2013) and *in vitro* and animal studies (see details in Carmeliet and Ruiz de Almodovar, 2013) report VEGF and PlGF stimulate multiple processes, including neurogenesis, enhanced neuronal survival, axonal growth, and migration and proliferation of glial cells.

Therefore, VEGFRs are excellent candidates to study to further understand the mechanism(s) of angiogenesis impairment and neural development in children born to preeclamptic pregnancies.

## VEGF Family and Preeclampsia

Preeclamptic women exhibit high plasma levels of sFlt-1 (Levine et al., 2004), and circulating levels of anti-angiogenic sFlt-1 in the fetoplacental circulation and children born to preeclamptic mothers are summarized in Table 1. Since the discovery of elevated sFlt-1 plasma levels in preeclamptic women, several studies have attempted to characterize the role of sFlt-1, PlGF, and VEGF in the pathophysiology of the condition (see details in Karumanchi, 2016, 2018), and the roles of these factors in preeclampsia are discussed below.

**Table 1:** Summary of findings of angiogenic factors in children born to preeclampsia in comparison with matched normotensive controls.

Age	Type of study	Sample size	Type of sample	Findings in preeclampsia	Reference
23–36 weeks of gestation	Cohort	123	Umbilical cord blood	VEGF was positively correlated, and sFlt-1 was negatively correlated, with birth weight and percentiles of weight for gestational age. Higher cord blood VEGF levels were associated with reduced risk of postnatal growth failure. The above biomarker associations were attenuated after adjustment for maternal preeclampsia.	(Voller et al., 2014)
30 weeks of gestation	Prospective	4108	Umbilical cord blood	High sFlt-1 (and reduced PlGF) were associated with reduced growth from the gestational stage to 6 years of age.	(Bergen et al., 2015)
Newborns at birth	Case-control	70	Umbilical cord blood	Elevated sFlt-1 in children of mothers with preeclampsia.	(Staff et al., 2005)
Newborns at birth	Cross-sectional	39	Umbilical cord blood	Lower VEGF levels and higher sFlt-1 levels.	(Olmos et al., 2013)
0–30 years	Prospective	204	Antecubital vein blood	Elevated levels of sFlt-1.	(Lewandowski et al., 2015)
5–8 years		43	Antecubital vein blood	No difference in sFlt-1 levels.	(Kvehaugen et al., 2011)

*sFLT-1: soluble receptor tyrosine kinase similar to fms-1; VEGF: Vascular endothelial growth factor; PlGF: Placental growth factor.*

### VEGF, sFlt-1 and Angiogenesis in the Offspring of Preeclamptic Pregnancies

Elevated levels of sFlt-1 have been reported in blood collected from umbilical cord (Staff et al., 2005; Tsao et al., 2005) and in adults (between 20 and 30 years old) originally born to preeclamptic pregnancies (Lewandowski et al., 2015). Also, studies have shown that neonates born from preeclamptic pregnancies exhibit lower levels of VEGF and higher levels of sFlt-1 when compared to children born to normotensive women (Olmos et al., 2013). In addition, it has been suggested that high levels of sFlt-1 in the umbilical cord are associated with impaired function of both endothelial cell progenitors (Xia et al., 2007) and human umbilical vein endothelial cells (Yu et al., 2016). Furthermore the impact of sFlt-1 on tissue function has been reported, and in the thyroid gland, a highly vascularized tissue, there is a negative association between thyroid hormone production and umbilical cord blood levels of sFlt-1 (Korevaar et al., 2014). Moreover, in the broader context of the systemic effects of sFlt-1 on infant growth and development, it has been reported that high umbilical levels of sFlt-1 are negatively associated with growth in infants in uncomplicated pregnancies (Bergen et al., 2015), and sFlt-1 levels are also negatively correlated with body weight percentile in preterm babies (Voller et al., 2014).

At the central nervous system level, the precise role of sFlt-1 in modifying neuroanatomical and vascular architecture, and circulatory characteristics in the brain is unknown. Interestingly, high circulating levels of sFlt-1 are associated with adult psychiatric and neurological alterations (Kim et al., 2007; Fulzele and Pillai, 2009; Emanuele et al., 2010; Lee et al., 2010; Lizano et al., 2016, 2017; Pillai et al., 2016) and studies also reveal a reduction in total frontal lobe volume in patients with schizophrenia/schizoaffective disorder (Pillai et al., 2016). Whether this association is the result of alterations in microvascular development in those patients is unknown, however, gene transfer of sFlt-1 in rats leads to a reduction in brain edema and in blood brain barrier permeability, suggesting a direct effect on brain endothelium (Kumai et al., 2007).

A limited number of studies have investigated the effect of sFlt-1 on brain development, including those of Carver et al. (2014) which report that, in a mouse model of preeclampsia, adenoviral transfer of sFlt-1 to the mother was associated with sex-dependent neuroanatomical alterations in the offspring at 6 months of age, which were partly counteracted by treating mothers with pravastatin (Carver et al., 2014).

If high levels of sFlt-1 in the mother or in the fetus effectively impair the proper development of brain blood vessels, and potentially brain development, in the offspring born from preeclamptic pregnancies (Figure 2B), sFlt-1 would represent a reliable prognostic biomarker that would help to predict adverse outcomes in children born from preeclamptic pregnancies.

### PlGF and Cerebral Circulation in Offspring of Preeclampsia

PlGF is highly expressed at all brain development stages (Luna et al., 2016) and is considered the key cerebral angiogenic factor (Liu et al., 2006; Gaal et al., 2013). Mechanistically, PlGF stimulates growth of neurons (Dewerchin and Carmeliet, 2012; Carmeliet and Ruiz de Almodovar, 2013) and formation of new blood vessels in the brain (Gaal et al., 2013) although the influence of PlGF on brain vasculature in preeclampsia has not been investigated.

However, studies report low levels of PlGF in maternal and fetal blood (Schlembach et al., 2007) and low levels of PlGF mRNA in the placenta (Andraweera et al., 2012) of preeclamptic pregnancies compared to normotensive pregnancies. Furthermore, low maternal levels of PlGF in the second trimester of gestation were associated with a narrower retinal arteriolar caliber (but not with a narrower retinal venular caliber) in childhood, compared to children who had experienced a normotensive pregnancy (Gishti et al., 2015). These findings suggest a highly selective role for PlGF in retinal vascular development.

A more direct relationship between PlGF levels during pregnancy and brain angiogenesis in offspring has been documented by Lecuyer et al. (2017), who demonstrated low placental levels of PlGF were associated with a reduction in radial distribution of fetal cerebral cortical microvasculature. Studies in normotensive mice virally transfected with PlGF (Gaal et al., 2013) and in PlGF (Plgf^−/−^) deficient mice (Freitas-Andrade et al., 2012; Luna et al., 2016) highlight the key role PlGF in the development of brain vasculature. Furthermore, seminal studies from Dr. Ana Croy’s group found that Plgf^−/−^ mice showed anatomical alterations in the cerebral macrocirculation at the level of anterior communicating cerebral arteries and anterior collateral vessels forming Willis’ polygon. These structural changes in cerebral circulation observed in Plgf^−/−^ mice were associated with impaired cognitive function (Luna et al., 2016), however, the latter study did not characterize the brain microcirculation, nor the profile of pro or anti-angiogenic factors in the Plgf^−/−^ mice. More recently, the same group has reported that PlGF^−/−^ mice also exhibited alterations in the development of neurons (Chaballe et al., 2011) and the retina (Kay et al., 2017).

## Is It All About Brain Angiogenesis?

Mechanistically, impaired brain angiogenesis is unlikely to be the unique alteration that fully explains the complexities of the adverse cognitive outcomes present in children born from preeclamptic pregnancies. Considering the hypothesis of fetal programming, it is feasible to speculate that other factors including oxidative stress, inflammation, neuronal structural modifications and epigenetic modifications driven by intrauterine hypoxia, among others, are involved. Furthermore, the contribution of many other post-natal factors, including socioeconomic environment, breastfeeding and social interactions to increase brain stimulation, to cognitive function in children born from preeclamptic pregnancies need to be established. Several excellent reports, including (Van den Bergh, 2011; Babenko et al., 2015) address the concept of fetal programming and alterations at brain level.

## Concluding Remarks

The syndrome of preeclampsia is a frequent complication of pregnancy, affecting both the mother and the fetus. This disorder is characterized by an imbalance between pro and anti-angiogenic factors released from the placenta and may lead to a series of adverse consequences in individuals throughout their post-natal life.

The evidence discussed in this review suggests that children of mothers with preeclampsia have a higher risk of developing cognitive disorders. However, despite increased research in this field, it is still unclear whether other confounding factors, such as intrauterine growth restriction, prematurity or asphyxia, contribute to the cognitive disorders in children born to preeclamptic pregnancies, and further studies are required to establish the underlying mechanisms responsible for such cognitive disorders. Herein, we present supporting evidence for alterations in the characteristics of brain microvasculature in the offspring born to preeclamptic women, although some studies include a small sample size that indirectly suggests structural alterations. While we highlight a potential key role of impaired brain vessel formation as an underling mechanism for cognitive alterations in children born to preeclampsia, it is also unlikely that this would be a unique mechanism for explaining the complex alterations in brain development and cognitive deficiencies observed. Furthermore, both inflammation and nutrient access (both over and undernutrition) represent potential contributory factors involved in brain alterations of offspring born to preeclamptic pregnancies.

We acknowledge that focusing on impaired VEGFR-mediated brain angiogenesis is an oversimplification of the complexity of the underling mechanisms of adverse cognitive outcomes in offspring born to preeclampsia. However, as indicated in this review, literature in this field is extremely limited and there is a substantial need for more focused research to establish the precise contribution of preeclampsia, independent of other confounding factors, on cognitive alterations in children. In particular, a better understanding of the roles of VEGF, PlGF, and sFLT-1 in impaired brain angiogenesis may aid in early diagnosis and management, and also in development of new treatment interventions.

## Author Contributions

EL wrote the draft of the manuscript. JA, JL, JP, and PT-V critically revised the manuscript. CE generated the original hypothesis and proposed the development of the review article.

## Conflict of Interest Statement

The authors declare that the research was conducted in the absence of any commercial or financial relationships that could be construed as a potential conflict of interest.
